# East African HIV care: depression and HIV outcomes

**DOI:** 10.1017/gmh.2019.6

**Published:** 2019-05-31

**Authors:** S. M. Meffert, T. C. Neylan, C. E. McCulloch, L. Maganga, Y. Adamu, F. Kiweewa, J. Maswai, J. Owuoth, C. S. Polyak, J. A. Ake, V. G. Valcour

**Affiliations:** 1 Department of Psychiatry, University of California, 401 Parnassus Avenue, San Francisco, CA, USA; 2 Department of Psychiatry, University of California, 4150 Clement St, San Francisco, CA, USA; 3 Division of Biostatistics, Department of Epidemiology, University of California, 550 16th Street, San Francisco, CA, USA; 4 Mbeya Medical Research Centre, P.O. Box 2410, Hospital Hill Rd, Mbeya, Tanzania; 5 U.S. Military HIV Research Program, Walter Reed Army Institute of Research, Abuja, Nigeria; 6 U.S. Embassy Nigeria, Plot 1075, Diplomatic Drive, Central District Area, Abuja, Nigeria; 7 Makerere University-Walter Reed Project, Plot 42, Nakasero Road, P.O. Box 16524, Kampala, Uganda; 8 U.S. Military HIV Research Program, Walter Reed Army Institute of Research, Silver Spring, MD, USA; 9 Henry M. Jackson Foundation Medical Research International, Hospital Road, P.O. Box 1357, Kericho, Kenya; 10 Henry M. Jackson Foundation for the Advancement of Military Medicine, 6720A Rockledge Drive, Suite 400, Bethesda, MD, USA; 11 U.S. Military HIV Research Program, 6720A Rockledge Drive, Suite 400, Bethesda, MD, USA; 12 Department of Neurology, Memory and Aging Center, University of California, San Francisco, CA, USA

**Keywords:** Depression, HIV, sub-Saharan Africa

## Abstract

**Importance.:**

Depression is a common co-morbidity for people living with HIV (PLWH) and is associated with elevated plasma HIV RNA levels. While depression correlates with deficits in antiretroviral (ARV) adherence, little data exist to inform the relationship between depression and HIV vial load more broadly.

**Objective.:**

To examine the relationship between depression and viral load in the African Cohort Study (AFRICOS) independently of ARV adherence.

**Design.:**

PLWH in Kenya, Uganda and Tanzania underwent screening for depression using the Center for Epidemiologic Studies Depression Scale (CESD) upon enrollment at AFRICOS HIV care sites.

**Setting.:**

AFRICOS is an ongoing prospective longitudinal cohort study enrolling HIV-infected adults at HIV care centers including sites in Kenya, Tanzania and Uganda. These sites are administered by President's Emergency Plan For AIDS Relief programs.

**Participants.:**

HIV+ individuals were eligible if they were at least 18 years old, receiving HIV care at the enrolling clinic and consented to data and specimen collection.

**Main outcome measure.:**

CESD.

**Results.:**

Among 2307 participants, 18–25% met the CESD threshold for depression. Depression was associated with decreased ARV adherence (OR 0.59, *p* =  0.01). Higher scores on three CESD items were significantly associated with 209–282% higher viral load, independently of ARV adherence among participants on ARVs ⩾6 months.

**Conclusions.:**

PLWH had high prevalence of depression on the CESD. Diverse depression symptoms were independently associated with increases in viral load, underscoring the need for comprehensive treatment of depression.

## Introduction

The strong association between HIV infection and depression is well established. In high-income countries, the estimated prevalence of depression among people living with HIV (PLWH) is approximately 25%, three times higher than HIV-uninfected individuals (Do *et al*., 2014). The proportion of HIV-infected individuals with depression may be even higher in low- and middle-income countries (Lowther *et al*., 2014) where mental health care is scarce (World Health Organization, 2016) and the majority of PLWH reside. Depression prevalence studies produce variable results, but reach as high as 63% among PLWH in sub-Saharan Africa (Bernard *et al.*, 2017).

### HIV viral load and depression: association with antiretroviral (ARV) adherence

One of the most common rationales for addressing depression in the setting of HIV is that depressed individuals are less likely to adhere to ARV regimes when compared with PLWH who are not depressed. The presence of depressive symptoms is associated with 42% lower likelihood of achieving ARV adherence (Uthman *et al*., 2014). Given the strong effect of ARVs on HIV viral load, adherence has been a primary focus for studies of depression among PLWH. This emphasis is reflected in mental health treatment studies with PLWH, where improved ARV adherence is often a primary objective of behavioral/psychosocial interventions. However, we may be unable to appreciate the more complex interactions between depression and HIV viral load, and, critically, opportunities to improve the overall mental and physical health of PLWH when a focus on the impact of depression on ARV adherence eclipses the other roles depression may play in the pathogenesis of HIV.

### HIV viral load and depression: associations that do not involve ARV adherence

Approximately 10 years ago, Ironson and colleagues found that high baseline depression symptoms predicted nearly a threefold increase of HIV viral load in the subsequent 2 years (relative to those with low depression scores at baseline), even after controlling for ARV adherence (Ironson *et al*., 2005). Since the publication of that study, support has been found for additional pathways between depression and viral load that do not involve ARV adherence, including cognitive and inflammatory pathways. However, much of research on the associations between depression and HIV viral load has focused on single mediators, such as cognition or ARV adherence, rather than including and controlling for multiple potential mediators.

### HIV and depression: cognition

HIV infection in the central nervous system (CNS) can lead to cognitive deficits among PLWH, termed HIV-associated neurocognitive disorder (HAND) (Fellows *et al*., 2013). The etiology of HAND is multifactorial and is thought to include the release of host's neurotoxic substances from infected CNS cells, exposure to HIV and its neurologically toxic components, cerebrovascular abnormalities, CNS inflammation (see below) and the toxic effects of ARVs. Depression, particularly major depressive disorder (MDD), also impacts neuropsychological testing performance (attention, learning, memory and processing speed) (McIntyre *et al*., 2013) and is associated with changes in the structure and function of fronto-subcortical brain regions and networks (Diener *et al*., 2012). Given the overlap between cognitive symptoms of depression and HAND and the high prevalence of depression among PLWH, there has been concern that HAND symptoms have been misclassified as cognitive symptoms of depression and vice versa (Hong & Banks, 2015).

### Research gap

While some HIV clinicians do assess for and treat mental disorders among their patients, the majority of PLWH and depression go without treatment, particularly in East Africa, where mental health care is scarce and HIV is highly prevalent. However, deficits in depression treatment for care-engaged PLWH in East Africa are modifiable. The field of global mental health care implementation research has advanced rapidly over the past decade, including the development of strategies to deliver integrated, high-efficacy, evidence-based depression treatment in East African HIV clinics using local, non-specialist personnel (Onu *et al*., 2016).

Extending mental health treatment to care-engaged PLWH in East Africa would benefit from greater clarity on how depression affects HIV viral load – a critical indicator for HIV clinicians. For example, if depression affects viral load primarily through ARV adherence, then depression treatment should focus on treatment engagement and correction of maladaptive health behaviors (such as non-adherence). On the other hand, if depression is associated with HIV viral load independently of ARV adherence, then other potential mediators (e.g. inflammation) may need to be more fully researched and depression treatment among PLWH may require a more comprehensive approach.

In this study, we address the research gap by examining the relationship between depression and viral load in a robust dataset from the African Cohort Study (AFRICOS) that allows us to control for adherence and cognitive performance.

## Methods

AFRICOS is an ongoing prospective longitudinal cohort study enrolling HIV-infected adults at 11 HIV care clinical sites in Kenya, Tanzania, Uganda and Nigeria. These sites are administered by five President's Emergency Plan For AIDS Relief (PEPFAR) programs (one per country except for two in Kenya: South Rift Valley and Kisumu West programs. In light of cultural differences in emotional expression within sub-Saharan Africa (Sweetland *et al.*, 2014), we restricted this study to AFRICOS sites in east Africa (Kenya, Uganda and Tanzania) and did not include the west African country of Nigeria. Specifically, we examined baseline data from East African HIV-infected AFRICOS participants (all of whom are engaged in HIV care) in Kenya, Tanzania and Uganda (*n* = 2307) to evaluate cross-sectional relationships between depression, cognition, HIV viral load and ARV adherence.

### Study design and participants

HIV-infected clinic clients were randomly selected to be study participants in a selection scheme that stratified by gender and ARV status and also included new enrollees to the clinic. A minority were recruited from other HIV studies performed by our group locally to facilitate long-term follow-up (*n* = 245).

Participant recruitment and enrollment for this study was between 21 January 2013 and 1 March 2017. HIV-infected individuals were eligible if they were at least 18 years old, receiving HIV care at the enrolling PEPFAR clinic and consented to data and specimen collection. We excluded individuals who were pregnant at enrollment.

### Procedures

Participants received a medical history and physical examination, a broad demographic and behavior questionnaire, and underwent phlebotomy. Depression symptoms were evaluated using the Center for Epidemiologic Studies Depression (CESD) scale, which was translated into Luganda, Luo and Kiswahili and performed as part of an interviewer administered questionnaire. All participants also performed a brief battery of neuropsychological tests (see below).

### Outcomes

#### Demographics

Participant age, gender and weekly household income were determined through chart review.

#### Income assessment across sites

*Purchasing power parity* (*PPP*). We calculated PPP of weekly household income to standardize and compare the cost of living across the three country sites. First, we converted local currency into 2016 USD dollars. We converted these figures to the market exchange rate (PPP), multiplying by 0.3 for Uganda and Tanzania and 0.5 for Kenya.

#### Depression

We assessed depressive symptoms using the Center for Epidemiologic Studies Depression Scale (CESD) (Eaton *et al*., 2004). The CESD is a 20-item measure that has been used extensively across high-, middle- and low-income populations to assess prevalence and severity of depression. Reponses are recorded on a four point Likert scale. The instrument has good validity and reliability with a Cronbach's *α* of 0.85–0.90 across studies (Radloff, 1977). A cutoff of 16 is typically used to indicate depression and has good sensitivity and specificity (Lewinsohn *et al*., 1997). The scale was translated and adapted using standard procedures including independent forward and backward translation, followed by pilot with test-subjects and incorporation of feedback. Due to variability in literacy required to complete the CESD, all items were read to study participants. *HIV viral load.* Viral loads (plasma HIV RVA levels) were measured using the on-site, Roche COBAS® Taqman® HIV-1 or Abbott RealTime HIV-1 Viral Load assays.

#### Treatment status

Participants were identified as being on ARV for all time-points following the date of first ARV prescription through chart review.

#### Adherence

ARV adherence was determined through self-report, using the following question: ‘In the past month, how many days did you miss a dose of your ARVs?’ One month recall of ARV adherence is associated with objective measures of adherence (see the ‘Limitations’ section) (Buscher *et al*., 2011). While we are aware that with modern ARV treatment, viral suppression can be achieved with only 80–84% adherence (Viswanathan *et al*., 2015), in light of emerging data showing that even in the setting of viral suppression, suboptimal (<100%) adherence correlates with significant clinical outcomes, including higher inflammation and residual viral replication (Li *et al*., 2014; Castillo-Mancilla *et al*., 2018*a*, *b*), we opted for a stringent definition of adherence, using a bivariate model assessing adherence over the past month in which 1 = no missed doses and 0 = doses missed.

#### Neuropsychological testing battery

All participants underwent a 30-min neuropsychological testing battery. Testers were trained on all neuropsychological tests, certified and re-certified every 6 months to assure consistent testing across all sites. The battery consisted of World Health Organization auditory verbal learning test (AVLT) trial 1 to assess attention, AVLT sum of trials 1–5 for learning efficiency and AVLT delayed recall for memory; Trails A to assess psychomotor speed; Grooved Pegboard to measure manual dexterity as well as psychomotor speed and action fluency to assess verbal performance. Normative data were acquired from 429 co-enrolled HIV-uninfected individuals who also sought to care at the same clinics and stratified to four levels of age and two levels of education. We calculated standardized *z*-scores in a standard fashion to identify degree of variance from the means of age and education matched strata. Cognitive impairment on neuropsychological testing was defined as at least −1 s.d. below the mean on two tests or −2 s.d. below the age and education matched mean on one test.

### Statistical analysis

We analyzed baseline characteristics of HIV-infected AFRICOS participants enrolled between 21 January 2013 and 1 March 2017, including depression and demographics by study site, as well ARV treatment status (on ARVs or not) and associations of key variables with depression. For those participants who had been prescribed ARVs, we evaluated the relationship between ARV adherence and depression using logistic regression to model ARV adherence over the past month as a bivariate, presenting the results as odds ratios (ORs) with 95% confidence intervals (CIs). Given skewness in residual analyses, we used bootstrapped (10 000 repetitions) linear regression to model the log_10_ HIV viral load among the sub-group of AFRICOS participants who had been on ARVs for 6 months or more. We included both past month adherence and a dichotomous measure of global cognitive impairment. We present the results as *β* coefficients with 95% CIs. Lastly, we conducted exploratory analyses of depression symptoms, by performing sequential bootstrapped (10 000 repetitions) linear regressions of log_10_ HIV viral load for AFRICOS participants who had been on ARVs for 6 months or more. Each CESD item for individuals was evaluated in a separate regression, controlling for ARV adherence and cognitive impairment. We present the results as *β* coefficients with 95% CIs. We also back-transformed regression estimates and CIs of log_10_ viral load to obtain interpretations as percent increase or decrease. Analyses were conducted using Stata/IC 15.0 (StataCorp, College Station, TX).

## Results

Most HIV-infected AFRICOS participants were from Kenya (63%), female (59%), middle aged (34–63 years old) (46%), married (59%) and taking ARVs (68%) at entry. Most reported household incomes of less than ½ international dollar per week. Literacy was high (89%), and the majority reported completing or partially completing primary school (Table 1). The percentage of AFRICOS participants engaged in HIV care meeting the CESD cutoff for depression: 18% (Kenya), 25% (Tanzania) and 22% (Uganda).

**Table 1.** Sample characteristics of HIV-infected AFRICOS adult participants at Kenyan, Tanzanian and Ugandan sites (*n* = 2307)

	Kenya *n* (%)	Tanzania *n* (%)	Uganda *n* (%)	Total *n* (%)
Totals	1444 (62.6)	388 (16.8)	475 (20.6)	2307 (100)
Taking ARVs				
Yes	1130 (78.3)	239 (61.8)	187 (39.4)	1556 (67.5)
No	314 (21.8)	148 (38.2)	288 (60.6)	750 (32.5)
Gender				
Female	832 (57.6)	231 (59.5)	282 (59.4)	1345 (58.3)
Male	612 (42.4)	157 (40.5)	193 (40.6)	962 (41.7)
Age				
18–33 years	388 (27.4)	122 (32.1)	146 (30.8)	656 (28.9)
34–48 years	658 (46.5)	153 (40.3)	227 (47.9)	1038 (45.8)
49–63 years	275 (19.5)	65 (17.1)	69 (14.6)	409 (18.0)
64+ years	13 (0.9)	14 (3.7)	5 (1.1)	32 (1.4)
Education				
Some primary school	496 (35.3)	40 (11.1)	246 (58.7)	782 (35.8)
Completed primary school	345 (24.5)	202 (56.0)	49 (11.7)	596 (27.3)
Some secondary school	198 (14.1)	37 (10.3)	105 (25.1)	340 (15.6)
Completed secondary school	259 (18.4)	56 (15.5)	5 (1.2)	320 (14.6)
Some university	38 (2.7)	3 (0.8)	0 (0)	41 (1.9)
Completed university	46 (3.3)	21 (5.8)	1 (0.2)	68 (3.1)
Completed vocational/training school	25 (1.8)	2 (0.6)	13 (3.1)	40 (1.8)
Ability to read and write	1362 (94.3)	347 (89.4)	356 (75.0)	2065 (89.5)
Marital status				
Single	174 (12.0)	79 (20.4)	34 (7.2)	287 (12.4)
Married	917 (63.5)	186 (47.9)	262 (55.3)	1365 (59.2)
Divorced/separated	134 (9.3)	44 (11.3)	134 (28.73	312 (13.5)
Widowed	219 (15.2)	79 (20.4)	44 (9.3)	342 (14.8)
Weekly household income in purchasing power^a^				
<0.5 international dollars/week	71 (4.9)	41 (11.3)	170 (35.3)	282 (12.2)
0.5–1.0 international dollars/week	89 (18.5)	31(8.5)	89 (18.5)	299 (13.0)
>1.0 international dollars/week	1209 (80.9)	291 (80.2)	223 (46.3)	1723 (74.8)

ARV, antiretroviral therapy.

a Income standardized across country sites to international dollars.

**Figure 1.**
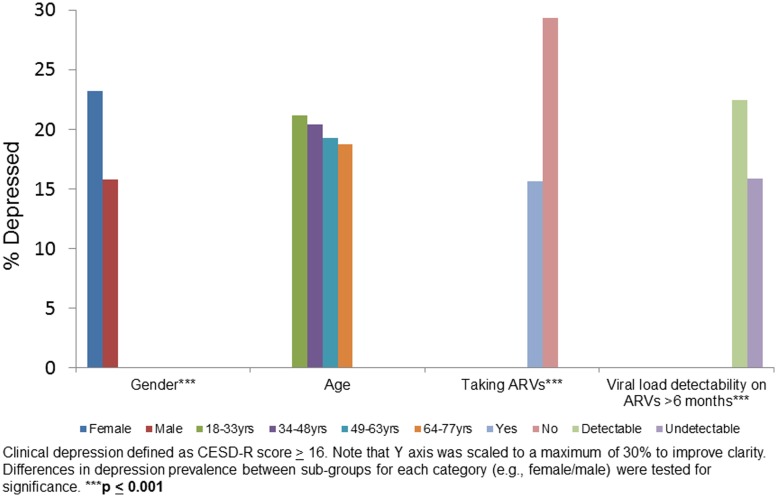
Clinical depression by gender, age, taking ARVs and HIV viral load suppression on ARV in baseline data: HIV+ participants (*n* = 2307).

We evaluated depression (percent with depression on the CESD) in sub-groups stratified by sex, age, ARV status and viral load on ARVs for 6 months or more (Fig. 1). Female sex (*p* < 0.001), not taking ARVs (*p* < 0.001) and detectable viral load (*p* < 0.001) were associated with higher proportion of depression relative to their counterparts in the same category.

Using logistic regression to model ARV adherence, we found that depression (bivariate) was inversely associated with ARV adherence (Table 2) for those individuals who were on ARV treatment (a subset of the total sample). Those with depression were nearly twice as likely to report adherence deficits in the past month, compared with their non-depressed counterparts (OR 0.59 [0.39–0.89], *p* = 0.012). Those with cognitive impairment were more likely to report full ARV adherence (OR 1.58 [1.09–2.27], *p* = 0.02).

**Table 2.** Logistic regression modeling ARV adherence over the past month (1 = no missed doses; 0 = doses missed) (*n* = 1400)

	OR [95% CI]	*p* value
Age	1.01 [1.00–1.03]	0.10
Education	1.02 [0.91–1.14]	0.70
Married	0.83 [0.59–1.18]	0.30
Weekly income converted to purchasing power^a^	1.01 [1.00–1.03]	0.13
Cognitive impairment (1 = impaired, 0 = not impaired)*	1.58 [1.09–2.27]	0.02
Depression (1 = CESD ⩾ 16 or 0 = CESD < 16)**	0.59 [0.39–0.89]	0.012

a Income standardized across country sites.

We used a bootstrapped linear regression to model log_10_ HIV viral load among individuals who had been on ARVs for 6 months or more (a subset of those on ARVs, Table 3). Covariates included ARV adherence and cognitive impairment. The log_10_ of viral load for those with depression had a 95% CI of −0.04 to 0.47, *p* = 0.10. This corresponds to a change in viral load of 0.91–2.95. Though not significant, the beta coefficient for depression was 0.21, which is a 60% increase in viral load.

**Table 3.** Bootstrapped multivariate linear regression modeling log_10_ viral load among HIV-infected participants on ARV for more than 6 months, controlling for past month ARV adherence and cognitive impairment (*n* = 1380)

	*β* [95% CI]	*p* value
Age	−0.01 [−0.02 to −0.01]	0.00
Education	−0.02 [−0.07 to 0.03]	0.43
Married	−0.10 [−0.27 to 0.06]	0.21
Weekly income converted to purchasing power^a^	−0.01 [−0.32 to 0.10]	0.31
Past month ARV adherence (1 = no missed doses, 0 = missed doses)	−0.44 [−0.71 to −0.17]	0.001
Cognitive impairment (1 = impaired, 0 = not impaired)	0.18 [0.01–0.35]	0.04
Depression (1 = CESD ⩾ 16, 0 = CESD < 16)	0.21 [−0.04 to 0.47]	0.10

a Income standardized across country sites; HIV viral load (VL).

### Item level analysis of the CESD

Controlling for ARV adherence and cognitive impairment, the log_10_ HIV viral load was modeled using separate bootstrapped linear regressions for each CESD item (Table 4). Associations were found with three CESD items (appetite, sleep and apathy/energy) of the CESD (#2 [*p* = 0.000], #11 [*p* = 0.01], #20 [*p* = 0.04]). Beta coefficients were 0.45, 0.32 and 0.32, respectively, corresponding to 282, 209 and 209% increases in viral load.

**Table 4.** Bootstrapped linear regression modeling log_10_ viral load on each CESD item (separately) among HIV-infected participants on ARV for more than 6 months, controlling for past month ARV adherence and cognitive impairment (*n* = 1477–1482)

	*β* [95% CI]	*p* value
Significant items		
I did not feel like eating; my appetite was poor (item #2)	0.45 [0.20–0.70]	0.00
My sleep was restless (#11)	0.32 [0.08–0.57]	0.01
I could not get going (#20)	0.32 [0.01–0.64]	0.04
Nonsignificant items		
I was bothered by things that usually don't bother me (item #1)	0.14 [−0.08 to 0.39]	0.20
I felt that I could not shake off the blues even with help from my family or friends (item #3)	−0.03 [−0.27 to 0.22]	0.84
I felt I was just as good as other people (item #4)	−0.17 [−0.35 to 0.01]	0.06
I had trouble keeping my mind on what I was doing (item #5)	0.08 [−0.22 to 0.41]	0.55
I felt depressed (item #6)	0.03 [−0.20 to 0.27]	0.77
I felt that everything I did was an effort (item #7)	0.08 [−0.05 to 0.20]	0.24
I felt hopeful about the future (item #8)	−0.06 [−0.26 to 0.15]	0.60
I thought my life had been a failure (item #9)	0.12 [−0.13 to 0.40]	0.32
I felt fearful (#10)	0.28 [−0.08 to 0.64]	0.13
I was happy (item #12)	0.08 [−0.13 to 0.28]	0.47
I felt hopeful about the future (item #13)	0.22 [−0.11 to 0.56]	0.19
I felt lonely (#14)	0.21 [−0.04 to 0.47]	0.10
People were unfriendly (#15)	−0.10 [−0.36 to 0.18]	0.51
I enjoyed life (item #16)	−0.10 [−0.25 to 0.06]	0.23
I had crying spells (#17)	0.12 [−0.23 to 0.49]	0.48
I felt sad (item #18)	0.20 [−0.02 to 0.42]	0.08
I felt that people disliked me (item #19)	0.01 [−0.31 to 0.35]	0.91

CESD items are scored on a 0–4 point scale (0 = rarely and 3 = most of the time), except for reverse coded items (#4, 8, 12, 16); HIV viral load (VL).

## Discussion

In this large sample of HIV-infected individuals engaged in treatment in HIV clinics in Kenya, Tanzania and Uganda, we found a high frequency of individuals with depression in samples from every country. Depression has been associated with care drop out (Krumme *et al*., 2015). Therefore, the proportion of participants with depression in this study may be lower than the prevalence among general populations of PLWH in the region. The prevalence estimate reported in this study could be viewed as the ‘tip of the iceberg’ with regard to the prevalence of depression among PLWH in these countries.

Women, individuals not taking ARVs, and those who had a detectable viral load on ARVs for 6 months or more, all had a higher prevalence of depression compared with their counterparts (Fig. 1). These findings are consistent with studies showing that depression is typically higher among women compared with men (Seedat *et al*., 2008; Albert, 2015) and associated with decreased ARV adherence and with increased HIV viral load (Ironson *et al*., 2005).

We evaluated the relationships between depression, ARV adherence and viral load, using regression models to assess for the effects of cognition. We found that when accounting for demographics and cognitive impairment, depression remained associated with a nearly 50% decrease in ARV adherence (Table 2). Cognitive impairment was unexpectedly associated with improved ARV adherence. Given that ARV adherence was assessed through participant recall and self-report of ARV adherence over the past month, it is possible that memory impairment may lead to recall errors and over-reporting of ARV adherence. When objective measures of ARV adherence are used, cognitive impairment is typically associated with poorer ARV adherence, relative to those without cognitive problems (Becker *et al*., 2011).

We examined correlates of HIV viral load, including ARV adherence, cognitive impairment and depression (Table 3), finding that depression had a near significant relationship with HIV viral load, independently of ARV adherence and cognitive impairment. Analyses with individual CESD items revealed that higher levels of three diverse symptoms (appetite, sleep and apathy/energy) were associated with viral load independently of ARV adherence and cognitive impairment – two of the three were associated with more than doubling of viral load and one with a near tripling (Table 4). While there were no CESD items that had indirect relationships with viral load, it may be that the composite CESD score is not significantly related to viral load because the effect of the three significant items is diluted by the addition of the 17 other items in the CESD total score calculation. We found that cognitive impairment was associated with higher viral load, consistent with literature (Tate *et al*., 2011).

### Somatic symptoms

Appetite, sleep and apathy/energy are diverse depressive symptoms, but they are all related to its somatic manifestations. Given the prevalence of somatic symptoms with chronic disease, it is possible that these symptoms do not reflect a mood disorder, but are simply the physical manifestations of HIV disease. The challenges associated with assessment for and diagnosis of depression in the setting of physical disease and chronic illness have been the topic of broader investigation for at least two decades. In 1997, Koenig and colleagues reported that inclusion of somatic depressive symptoms when diagnosing major depression among medically ill patients resulted in high sensitivity for diagnosis, whereas excluding somatic symptoms led to higher specificity, but missed nearly 50% of MDD cases (Koenig *et al*., 1997). Currently, the emerging model is one of reciprocal relationships between physical and mental disease in which each increases the risk for the other, coupled with greater understanding depression as ‘systemic’ disease reflecting inflammatory, hormonal and many other disturbances (Katon, 2011; Thom *et al.*, 2019). It is also important to note that somatic symptoms are more common among depressed individuals who reside outside Euro-America, suggesting that a diagnostic approach that does not discount somatic symptoms of depression may be even more prudent for the study's target population (Lacasse *et al*., 2014; Evagorou *et al.*, 2016; Dreher *et al*., 2017). Finally, unlike most studies of depression in the setting of physical disease, we included cognitive impairment in our models given concerns that cognitive symptoms might reflect the direct effects of the HIV virus on the CNS rather than depressive symptoms. While benefitting from the analyses in many ways, it is possible that this decision also excluded individuals who were experiencing cognitive deficits secondary to depression and not HIV infection, reducing the strength of the relationship between viral load and depression in the first analyses (Tables 2 and 3) and potentially obscuring the relationship between HIV viral load and non-somatic CESD items (Table 4). Thus, the findings from this study should be understood as identifying depressive symptoms which remain associated with key HIV outcomes such as viral load even with quite conservative assumptions as to the source of the symptoms. Findings should not be interpreted as indicating that somatic symptoms are the only relevant depressive features for PLWH in the target population.

Indeed, one of the key findings of this study is that a number of depressive symptoms on the CESD are associated with HIV viral load independently of ARV adherence and cognitive impairment. These results suggest that, even if the effects of depression on ARV adherence could be fully addressed through behavioral adherence strategies and HIV-related cognitive deficits remediated, individuals with depression, particularly those with low appetite, poor sleep and apathy/energy, would still have an increased HIV viral load.

## Limitations

This was a cross-sectional study of baseline data from a longitudinal study. The results cannot be used to determine causality. Future research will leverage the longitudinal nature of this study to assess the predictive value of depression on critical HIV outcomes.

### CESD

We note recent analyses of depression in HIV-infected populations that question the validity of the CESD when used with heterogeneous samples of diverse genders (including transgender) and racial groups (*n* = 347) (Gay *et al*., 2016). Given the larger sample size and greater homogeneity in this East African, care-engaged study population, we believe the results to be a valid indication of the associations among depression, HIV viral load and cognition. Furthermore, the CESD uses self-report of depressive symptoms, rather than clinical interview. While the CESD is found to correlate adequately with clinical interview in many studies (e.g. Shinar *et al*., 1986; Irwin *et al.*, 1999; Moon *et al*., 2017), there are some studies in which it is found to have a significant number of false positive diagnoses (Quiñones *et al*., 2016; Vilagut *et al*., 2016). If the CESD in this study over-estimated depression, then we would expect the actual depression prevalence to be lower than reported. Furthermore, if the CESD results contained false positives, the relationship between depression and HIV viral load might be under-estimated by these data. Given ongoing disagreement regarding the validity of the CESD, we recommend that validity studies be undertaken in which CESD results are compared with a gold-standard, culturally accepted structured clinical interview, such as the Mini International Neuropsychiatric Interview (MINI), Major Depressive Disorder module and clinical exam for other specified depression and unspecified depression.

### Confounders

The number of confounders of the relationship between depression and HIV viral load that could be included in this study was limited by the study design, determined before our engagement with the AFRICOS team. Confounders are defined as variables that influence both the dependent and the independent variable(s), creating the appearance of an association. In particular, while alcohol and other substance abuse can be related to ARV adherence and mood disorders, we selected not to include it in our analyses for the following reasons. Most analyses of the relationships between depression and substance abuse have found the interaction to be bi-directional. Furthermore, studies now advise that co-occurring substance use disorders, do not change the recommendation to treat depression with evidence-based care (Pettinati *et al.*, 2013; Baingana *et al*., 2015; Tolliver & Anton, 2015). Therefore, while the presence of substance use disorder(s) may require the addition of mental health services, it does not typically change the clinical imperative to treat depression. Given the traumatic experiences of many PLWH in sub-Saharan Africa, posttraumatic stress disorder (PTSD) is a common co-morbidity. While measures of PTSD were not initially included in AFRICOS, during the course of our work, we were able to add a measure to the AFRICOS assessment schedule and look forward to evaluating the findings and the relationships between PTSD, depression and viral load.

### ARV adherence and cognitive impairment

AFRICOS uses a self-report measure of ARV adherence. Self-reported adherence is known to be subject to recall and social desirability bias, which can lead to inflated reports of adherence (Castillo-Mancilla & Haberer, 2018).

Our analyses of the cognitive battery deployed a binary assessment of impairment/non-impairment. This decision did not capitalize on data for individual cognitive domains produced by the study's cognitive battery and thus could have resulted in some loss of precision. However, given that our goal with the cognition variable was to control for the potential overlap in cognitive deficits seen in both HIV CNS infection and depression, coupled with the lack of current consensus on the precise effect of either depression or HIV on specific cognitive function domains, we felt that the summary measure was the most inclusive and comprehensive approach.

## Conclusions

This study supports the idea that even treatment-engaged PLWH in sub-Saharan Africa with PEPFAR-supported care may frequently suffer from depression. We provide evidence that diverse depressive symptoms may be associated with viral load independently of pathways involved with cognition and ARV adherence – implying that comprehensive depression treatment would be necessary to fully address the relationship between HIV viral load and depression. Given the recent advances in mental health care delivery using local non-specialists in sub-Saharan Africa and PLWH, key next steps include research on scalable, evidence-based depression treatment with careful assessment of impact on HIV outcomes.
